# Fragmented but evolving: a response to the editorial “the Italian health data system is broken”

**DOI:** 10.1016/j.lanepe.2025.101256

**Published:** 2025-03-04

**Authors:** Francesco Andrea Causio, Davide Golinelli, Giacomo Diedenhofen, Andrea Silenzi, Diana Ferro, Francesco Baglivo

**Affiliations:** a Italian Society of Artificial Intelligence in Medicine (SIIAM, Società Italiana Intelligenza Artificiale in Medicina), Rome, Italy; b Section of Hygiene, University Department of Life Sciences and Public Health, Università Cattolica del Sacro Cuore, Rome, Italy; c Department of Life Science, Health, and Health Professions, Link University, Rome, Italy; d Specialization School in Medical Statistics and Biometry, Department of Public Health and Infectious Diseases, Sapienza University of Rome, Rome, Italy; e Center for Research and Studies on Leadership in Medicine, Università Cattolica del Sacro Cuore, Rome, Italy; f Predictive and Preventive Medicine, Bambino Gesù Children Hospital, IRCCS, Rome, Italy; g Department of Translational Research and New Technologies in Medicine and Surgery, University of Pisa, Pisa, Italy

We read your recent editorial^1^ with great interest and would like to thank the editors for highlighting critical issues within the public, single-payer Italian National Health Service (SSN) data systems. However, we believe that the assertion that the “Italian health data system is broken” oversimplifies the complexities of an evolving system that, despite its criticalities, holds significant strengths and the potential to develop into a unified, efficient, and forward-thinking healthcare data ecosystem.

Italy's 21 regions and autonomous provinces, with their federalized healthcare systems, do indeed experience fragmentation and heterogeneity in their health data systems. However, it should be noted that this issue does not make Italy an isolated case in the European context. Nonetheless, the SSN possesses a significant backbone of unified and centralized clinical and administrative data flows, based on standardized data collection and transfer protocols. These include inpatient and outpatient care services, pharmaceutical services, and others, all currently interlinked at the individual level and unified at the national level in the “New Healthcare Information System” (NSIS), covering approximately 85% of all national benefits package services.^2^ This expanding system represents an important step towards a fully integrated and digitized national health information system.

The Italian Electronic Health Record (Fascicolo Sanitario Elettronico - FSE) system is also integral to this process. In Italy, the nationwide adoption of the FSE began in 2012, building on existing regional initiatives. Today, the FSE system is still regionalized, with varying usage rates among citizens and practitioners (Fig. 1).^3^ Yet the 2024 Digital Decade study on eHealth data accessibility for citizens considers Italy a “fast tracker” in the EU-27, with an above-average maturity score and significantly improved performance year on year.^4^ As of November 2023, 57,663,021 FSEs were active, covering 97·8% of the Italian population, and 94% (42,016) of all general practitioners accessed the FSE system in Q3 of 2024.^3^ Italy's Recovery and Resilience Plan (PNRR) is funding reforms in healthcare data systems, telemedicine, and digitalization, including the “FSE 2·0”, which builds on existing data infrastructures with the goal of ensuring full FSE interoperability between regions by 2026, and provide citizens with a comprehensive repository of their natively digital health data.

**Fig. 1:**
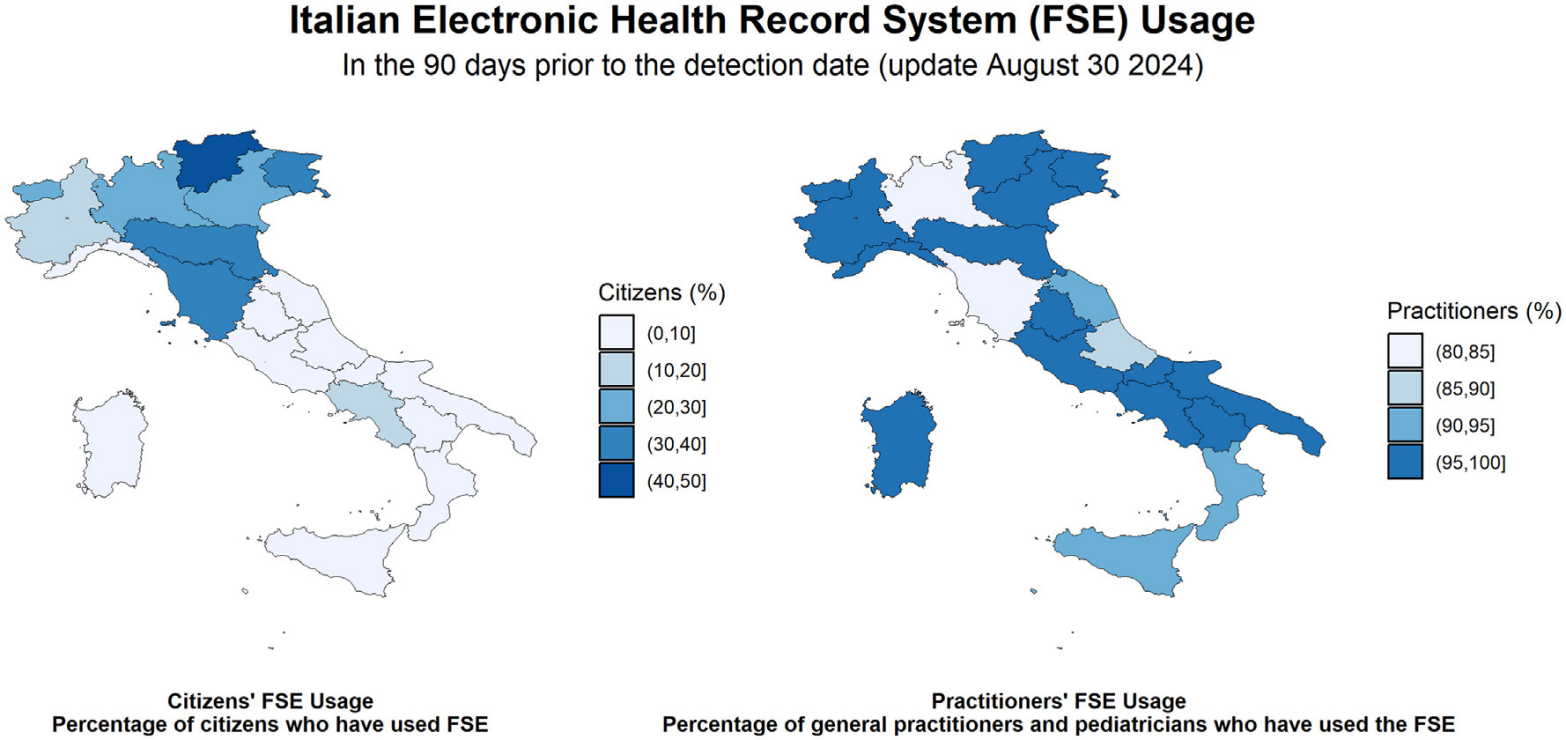
Proportion of citizens and healthcare practitioners utilizing the Italian Electronic Health Record System (FSE) across regions. Data represents the 90 days before August 30, 2024. For the Trentino–Alto Adige region, data from the autonomous healthcare systems of the provinces of Trento and Bolzano are reported as an average. Fig. 1 was crafted using publicly available data (Ministry of Health, Italy. *Fascicolo Sanitario Elettronico usage data*. Available from: https://monitopen.fse.salute.gov.it/usage - accessed Jan 6, 2025) and R version 4.2.2, with the libraries sf, ggplot2, and patchwork.

Paired with the capillary network of primary care providers and specialists, these developments aim for a comprehensive and interoperable digital health system that follows citizens along their health journey, provided that adequate policy allows for data access, transfer, and elaboration. Here, too, we find reasons to be optimistic, as recent legislative developments, such as the revised Privacy Code, also facilitate secondary health data use and have aligned Italy more closely with European standards.^5^

Italy has a rich legacy of scientific and medical expertise, and a wealth of health data from which to draw. With the right policies and investments, it has the potential to become a leader in health data innovation. This requires a collective effort to balance local autonomy with national cohesion, ensuring that technological advancements are shared and implemented effectively across regions while agreeing on open data frameworks and interoperability. To succeed, this evolution must involve policymakers, healthcare professionals, and researchers, ensuring equity and public trust remain central. The challenges in harmonizing systems and standards are evident, but they also represent an opportunity to leverage innovation and drive the systemic changes needed to unlock the full potential of Italy's health data system.

## Contributors

FAC and FB conceptualized the article. All authors reviewed literature, wrote the first draft, reviewed, and provided edits. FB performed data visualization. All authors validated the final version of the article.

## Declaration of interests

The authors declare no conflict of interest.
